# Deaths at Five, A. M.

**Published:** 1872-04

**Authors:** 


A YOUNG MAN in Hartford read somewhere that more deaths occurred at five o'clock in the morning than at any other hour, and now gets up regularly at four, in order to be out when Death makes his morning calls.

